# Implicit short- and long-term memory direct our gaze in visual search

**DOI:** 10.3758/s13414-015-1021-3

**Published:** 2016-01-11

**Authors:** Wouter Kruijne, Martijn Meeter

**Affiliations:** Vrije Universiteit Amsterdam, Amsterdam, Netherlands

**Keywords:** Visual search, Priming, Eye movements, Long-term memory, Implicit memory

## Abstract

Visual attention is strongly affected by the past: both by recent experience and by long-term regularities in the environment that are encoded in and retrieved from memory. In visual search, intertrial repetition of targets causes speeded response times (*short-term priming*). Similarly, targets that are presented more often than others may facilitate search, even long after it is no longer present (*long-term priming*). In this study, we investigate whether such short-term priming and long-term priming depend on dissociable mechanisms. By recording eye movements while participants searched for one of two conjunction targets, we explored at what stages of visual search different forms of priming manifest. We found both long- and short- term priming effects. Long-term priming persisted long after the bias was present, and was again found even in participants who were unaware of a color bias. Short- and long-term priming affected the same stage of the task; both biased eye movements towards targets with the primed color, already starting with the first eye movement. Neither form of priming affected the response phase of a trial, but response repetition did. The results strongly suggest that both long- and short-term memory can implicitly modulate feedforward visual processing.

**Electronic Supplementary Material:**

The online version of this article (doi:10.3758/s13414-015-1021-3) contains supplementary material, which is available to authorized users.

Since the visual environment is too rich for our visual apparatus to fully process, we are constantly confronted with the problem of which parts of a visual scene we must select for further processing, at the expense of other information. The mechanisms and processes by which such selection takes place are collectively known as visual attention. Research on visual attention has traditionally subdivided influences on attention into two classes: attention can be driven ‘bottom-up’ by the physical properties of the visual input and ‘top-down’ by factors such as our current goals and intentions. The main focus of the study of visual attention has long been the interplay between these two classes, and the question to what extent one can overrule the other (Chun et al. 2011; Van der Stigchel et al. 2009).

However, this extensively studied dichotomy does not seem to cover all factors that affect visual information processing. For example, whether we are navigating a busy street or taking a calm walk in the forest, it is rarely clear-cut what the top-down goal is of ongoing visual processing. Nevertheless, continuously allocating attention to—and only to—conspicuous features in the scene might not be the safest, most effective or most relaxing strategy (see Anderson et al., (2015), on the limits of salience effects in scene viewing). Recently, researchers have began to emphasize that attention for a large part may not be driven by our ‘top-down’ goals or the ‘bottom-up’ visual input, but is guided automatically and implicitly by our memories of past, similar experiences (Hutchinson and Turk-Browne 2012; Awh et al. 2012; Peelen and Kastner 2014).

An example from experimental psychology illustrating how memory affects visual attention is formed by *intertrial priming*, a phenomenon that was first thoroughly explored by Maljkovic and Nakayama (1994). In their experiments, participants searched for a red or green singleton diamond among two diamonds of the opposite color, and responded to an orthogonal feature. Repeating target- and distractor colors evoked shorter response times (RTs) compared to color switches. Perhaps one of the most interesting characteristics of priming that was found by Maljkovic and Nakayama (1994) was that priming is also found when a color switch is fully predictable or when participants are informed before trial onset, which illustrates that such priming is not top-down controlled (see also Huang et al., 2004; Hillstrom, 2000; Theeuwes and Burg, 2011; Theeuwes, 2013).

Priming has been found to manifest so early that it can modulate visual signals before they reach the oculomotor system (Meeter and Van der Stigchel 2013; Bichot and Schall 2002). On the other hand, priming seems to similarly affect later stages of a search task, such as the selection of a response (Lamy et al. 2010; Tollner et al. 2008; Meeter and Olivers 2006; Kristjánsson et al. 2008). Perhaps because of these diverse findings, research has not pinpointed a single mechanism to account for priming in visual search. Rather, two mechanisms have been proposed to contribute to these effects: (1) temporarily changes in the abstract ‘weights’ of target and distractor features (Maljkovic and Nakayama 1994; Maljkovic and Martini 2005; Lee et al. 2009), and (2) automatic and implicit retrieval of memory traces of past trials, facilitating search when they match present experience (Hillstrom 2000; Huang et al. 2004).

Most recent accounts of priming acknowledge a contribution of both these mechanisms, but argue that priming of low-level perception is dominated by the weighting of features, and that the retrieval of memory traces has effects on later stages (Huang et al. 2004; Lamy et al. 2010). Additionally, it has been argued that the effects of retrieval are limited to difficult tasks (Lamy et al. 2011; Ásgeirsson and Kristjánsson 2011).

Regardless of its underlying mechanisms, priming is commonly viewed as a short-term memory effect (Martini 2010; Maljkovic and Nakayama 2000; Kristjánsson and Campana 2010; Lee et al. 2009; Hillstrom 2000), as the effects of a single trial subside over the course of 5–8 trials (Maljkovic and Nakayama 1994; Martini 2010). In pop-out search, color repetitions further back do not seem to affect priming, however many that may be (Maljkovic and Martini 2005; Kruijne et al. 2015), although see (Geyer and Müller 2009). Even in the absence of intervening trials, priming attenuates with time, seemingly fading over 90 s; (Maljkovic and Nakayama 2000; Thomson and Milliken 2012). However, this assessment—that priming would only be short-lived—would set it apart from most other effects of memory on visual attention found in the literature (Turk-Browne et al. 2010; Chun and Jiang 1999; Leber et al. 2009), which are often acquired and assessed over the time course of entire experiments. Also, if priming is indeed to some extent driven by retrieval of memory traces of past trials, there is little reason that this would be limited to retrieving only recent experience.

In recent experiments, we investigated whether priming could also manifest itself over longer time ranges (Kruijne and Meeter 2015). In a set of visual search experiments, two possible target colors randomly alternated across trials, which resulted in intertrial priming. Critically, one target type was presented more often than the other in some blocks. This resulted in *long-term priming*: a prolonged facilitation of RTs for this biased color (Fig. 1a). This effect robustly persisted for at least 200 trials after the bias was removed, and was similar in participants who were and who not aware that there had been a color bias.

**Fig. 1:**
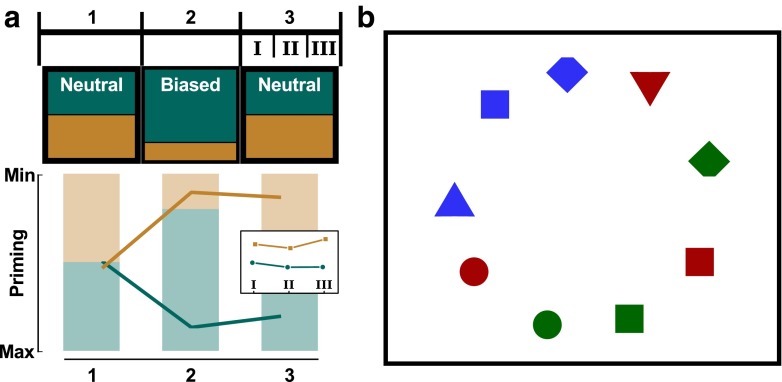
**a** The experimental design (up to block 3) of the experiments used to investigate long-term priming. The *colors* indicate the trial type distribution in each block. This was equal for both targets in Neutral blocks, but 80 % of targets were ‘bias-colored’ during Biased blocks. Long-term priming is defined as speeded RTs for biased-color targets during neutral blocks induced by this bias. Sub-blocks I–III were defined to explore long-term priming at a finer time scale. The *bottom graph* depicts this effect (not real data). **b** Schematic illustration of a search display as used in this study. The task is to search for a red or green diamond (see Methods for further detail)

Interestingly, long-term priming was only found when participants searched for a conjunction target. The same manipulation in singleton search tasks did not yield any long-lasting effects, even when search was rendered inefficient. Because of this strong dissociation, we proposed that different priming mechanisms dominate in both types of search: whereas priming in pop-out search may be dominated by short-lived feature weighting as described by Maljkovic and Nakayama (1994), priming in conjunction search would be largely driven by the retrieval of memory traces—in particular by those formed recently or frequently, resulting in short- and long-term priming.

Note that in this proposal, short- and long-term priming in conjunction search are both caused by the same mechanism, which would imply that they affect visual search performance in a functionally similar fashion. Using only response times, however, we could not uncover whether this is indeed the case, or whether short- and long-term priming actually manifest at very different stages of visual search. Various proposed theories of priming would predict that this is indeed the case. Huang et al. (2004) explicitly stated that priming from retrieving past trials operates like a sort of a ‘checking’ mechanism that is engaged *after* the target has been found. Lamy et al. (2010, 2011) extended this idea by suggesting that priming is a composite effect of a perceptual stage, which is always facilitated in the case of target repetition, and a post-perceptual stage where response repetition and target repetition interact. Since (Huang et al. 2004), such interactions have been taken to reflect the retrieval of previous trial traces. This would operate through the retrieval of the past trial episode, where responses are facilitated only if both the response and the target feature match.

Motivated by the support for multistage accounts of priming (Meeter and Olivers 2006; Tollner et al. 2008; Ásgeirsson and Kristjánsson 2011), and by the hypothesis that the retrieval-based component to priming only affects search performance after the target has been found, we investigated whether long- and short-term priming affect different stages of a search trial. We did so by deriving measures from eye movement recordings during search that could reflect different stages of the task. Although eye movements do not directly measure covert visual attention (Posner 1980; Pashler et al. 2001), they are tightly coupled (Rayner 2009; Hoang Duc et al. 2008; Zelinsky and Sheinberg; Sheliga et al. 1994); research suggests that eye movements cannot be made without a preceding covert attentional shift (Schneider and Shiffrin 1977; Zhao et al. 2012; Kowler et al. 1995). Consequentially, eye movements have often been employed as a tool to study visual search.

Previous research found that the effects of priming on response times are reflected in eye movements during search (Becker and Horstmann 2009; Bichot and Schall 1999a; McPeek et al. 1999). Becker and Horstmann (2009) found that in a conjunction search task, the eyes were biased to land on items that shared features with the previous target, but that the time between landing on a target and responding was not affected. From this, they inferred that intertrial priming in conjunction search relies on feature weighting.

Here we extend this approach by investigating how long-term priming affects the search process. Here, too, we looked at both the effects of priming on early search—defined as an effect on where the first saccade is targeted—and an effect at the response stage—defined as a shortening of the time in between the eyes landing on the target, and the response being elicited. As a manipulation check, we also investigated straight response priming, in which responses that are repeated are speeded. Such priming should affect the response stage as defined above, which was indeed what we found. To preview our other findings, we found that long-term priming had the same characteristics as shortterm feature priming: it affected the search process early on, but not the response phase. Additionally, we explore how other biases affect search behavior, such as positional priming and idiosyncratic location biases.

## Methods

### Participants

Participants were 25 students from the Vrije Universiteit Amsterdam (24 female, aged 18–29, M = 21.3, SD = 3.1). All reported normal color vision, and otherwise normal or corrected-to-normal vision. They were naive with respect to the purpose of the experiment or the trial imbalance manipulation. Participants received course credits or monetary compensation. Informed consent was obtained prior to the experiment, in accordance with the guidelines of the Declaration of Helsinki. Because all participants performed well over 90 % correct and no participant had average RTs more than 3 standard deviations away from the group mean, no participants were excluded from the analyses.

### Materials

Stimuli were presented on a 22-inch LCD monitor at 1680×1050 resolution and a refresh rate of 120 Hz. Participants were seated at 70-cm distance in a dimly lit room, and used a chin rest to maintain their head in place. Their left eye was recorded using an Eyelink 1000 eye-tracker (SR-research), which has a 0.01 ^∘^ resolution and 0.25 ^∘^ – 0.50 ^∘^ accuracy; The sampling rate was 1000 Hz. The built-in methods of the Eyelink were used to identify fixations, blinks, and saccades (at 35^∘^/*s* velocity and 9500^∘^/*s*^2^ acceleration threshold).

### Stimuli and procedure

The experiment was programmed using OpenSesame with the PsychoPy back-end (Mathôt et al. 2012; Peirce 2007). Stimuli and procedure were virtually identical to that used in (Kruijne and Meeter 2015, Experiment 2B), the only difference being that set size was not varied but fixed at nine items. A schematic example display is depicted in Fig. 1b. All items were red, green, or blue (approximately matched in luminance: 12.8, 13.3, and 13.1 cd /*m*^2^, respectively) and had one of four primitive shapes: diamonds pointing up or down, circles, squares, and triangles. Every display contained one red or green diamond (the target), one blue distractor diamond, two blue non-target shapes, and five red or green non-target shapes, all randomly assigned but chosen to balance the colors and shapes within each display. Each diamond spanned 1.33^∘^ of visual angle, and was missing a corner at the top or the bottom by covering up 1/8 of the diamond height. The other shapes were sized to match the diamond in surface area.

Twenty-four possible stimulus locations were identified at equidistant locations on an imaginary circle at 5.4^∘^ eccentricity. On each trial, the nine stimuli were randomly distributed over these locations, though never immediately adjacent to another. As a result, the minimal arc between two stimuli was 30 ^∘^, and the maximum arc 120 ^∘^.

Every trial started with a small fixation cross that participants were to fixate and press the space bar for drift correction. This cross was then followed by a fixation dot for 1200-1700 ms, after which the search display appeared. Participants were instructed to search for the red or green diamond in the display, and indicate on a keyboard which of its corners was missing, by pressing ‘U’ for up and ‘D’ for down. They were encouraged to respond as fast as possible while maintaining over 90 % accuracy.

The experiment started with ten practice trials, and then consisted of five blocks of 200 trials, with (three) Neutral and (two) Biased blocks alternating. In Neutral blocks, both red and green were the target color equally often, and the number of intertrial repetitions and switches was closely matched (<5 *%* difference). In both Biased blocks, the same color was the target on 80 % of the trials (red and green counterbalanced across participants). To the participant, the experiment consisted of eight segments of 125 trials, separated by small, self-timed breaks. This produced an asynchrony between breaks and block limits to prevent participants from easily identifying neutral and bias blocks and use a different strategy in them.

In our previous work, we found that the long-term priming effect was robust across a neutral block, and it did not attenuate over the course of 200 neutral trials. This constituted strong evidence that long-term priming is not the mere consequence of cumulative short-term priming effects (Maljkovic and Martini 2005; Martini 2010). To assess the evolution of the long-term priming effect during a neutral block, we divided each neutral block into three sub-blocks (I, II, III) of 66 or 67 trials.

At the end of the experiment, participants’ subjective experience of the color bias was assessed. They were presented with a line with tick marks at both ends and in the center, which were labeled ‘only red targets’, ‘equal amounts of red and green targets’ and ‘only green targets’. By drawing a mark on this line, they indicated on a continuous scale what they would estimate their target color distribution had been.

### Data processing and dependent measures

Trials with incorrect responses were excluded from the analyses unless noted otherwise, as well as trials immediately following a break. To identify outliers, we determined the mean and standard deviation of all trials in the neutral blocks, and then excluded all trials from all blocks where RTs that were more than three standard deviations away from the mean from further analyses. On average, this led to the exclusion of 4.3 % of trials, of which 2.9 % due to being incorrect and 1.4 % outliers (total exclusion rate 1.2–9.1 % across participants).

For all univariate measures (see ‘Analyses’ below), we only assessed the data from neutral blocks that followed a bias block, i.e. from Block 3 and Block 5. Of primary interest was how priming affected RTs. As in our previous work, of interest for long-term priming is how the relative search performance for either target color changes in response to the bias blocks, and this we wanted to correct for any a priori color biases. However, overall RT tends to decrease over the course of an experiment due to practice, which is likely to affect the magnitude of any effects on reaction time (see e.g., Martini, 2010) To overcome this, we first applied a z-transform ($zRT =\frac {RT-M}{SD}$). The z-scores in all blocks were then adjusted for a priori color difference by subtracting the zRT difference between both colors in the first block, which preceded the color bias.

The same correction procedure was applied to all dependent measures identified below, unless stated otherwise. The figures in the main text will mostly depict absolute, uncorrected measures in all blocks, to report the overall performance on the task. Whenever omitted from the main text, graphs of color-corrected values as used in the statistical analyses are provided as supplementary figures.

Accuracy was analyzed for priming effects, but since participants were instructed to maintain high accuracy, no effects were expected.

Two measures were derived from the fixation events (as identified by the eye tracker) to investigate how priming influences *search*: the average number of fixations detected in a trial (excluding the first, central fixation), and the color of the item that was first fixated after display onset. Two other measures were derived to investigate how priming may affect *response production*, after the target is found: the time between the last fixation on the target and the response, and the total duration of all fixations on the target during a trial. For these analyses, we define that the gaze was ‘on’ a stimulus when it was less than 2^∘^ away from the center, and no item is closer.

To further explore search behavior at a more fine-grained temporal resolution, we employed an event-related design comparing gaze samples at time points relative to display onset and response. Epochs were defined from -50 to 600 ms and -750 to 0 ms relative to display onset and the response, respectively. In these epochs, at every millisecond the proportion of samples on a particular item color were compared.

### Analyses

All univariate measures were analyzed with a Bayesian analysis of variance (ANOVA) using the BayesFactor package (Rouder and Morey 2012; Rouder et al. 2012) in R.

The ANOVA included ‘participant ID’ as a random effect, and the following factorial terms: short-term priming (*S*, immediate target repetition or switch); long-term priming (*L*, target type, bias colored target or other color); response priming (*R*, immediate target response repetition or switch); and variation of effects across sub-blocks (*B*), a term primarily included to assess whether long-term priming seemed to decay within a neutral block (which would be found as an *L*×*B* interaction).

The statistic of interest in the Bayesian ANOVA is the Bayes Factor (*BF*_*x*,*y*_), which describes the relative likelihood of two models ($\mathcal {M}_{x} $ and $\mathcal {M}_{y}$) given the data. A *BF*_*x*,*y*_=3 implies that the observed data are three times more likely to have occurred under $\mathcal {M}_{x} $ than under $\mathcal {M}_{y} $, and that BF$_{y,x}=\frac {1}{3}$. Exact Bayes Factors up to 1000 are reported.

Computing the *BF* across all possible models that arise in this 2×2×2×3 design is computationally unwieldy. Therefore, the univariate measures were assessed using the same ‘top-down’ model comparison procedure^1^. In this procedure, the evidence for every term *k* (which can be either a main effect or interaction term) is assessed by comparing the full model $\mathcal {M}_{F}$ versus a model excluding the term $\mathcal {M}_{F-k}$. This comparison yields a *BF*_*k*_ = *x* with *x* denoting the evidence *against* removing a term from the full model. All univariate measures (RT, number of fixations, proportion of first fixated color, target fixation time and total target fixation time) were analyzed via the same 2×2×2×3 ANOVA.

As a general rule, only those terms with positive evidence (*BF*_*k*_>1) are reported. For these terms, we also report the mean (M) and 95 % confidence interval (CI) of effect sizes. These are computed from the posterior distribution estimate of the differences between factor levels, derived from 5×10^4^ Markov Chain Monte Carlo (MCMC) samples.

Because we primarily sought to compare the factors underlying long- and short-term priming effects, we planned to test participants until the analysis showed strong evidence for—or against—an effect of these terms on RT at *BF* > 10.0 (see Wagenmakers et al., (2012, 2010), for justifications of this approach). This criterion was already met after data were collected from a first cohort of 25 participants.

The multivariate time-series analysis of the proportion of samples on either color was separately conducted for both forms of priming (short and long term), for both the display-locked epoch and for the response-locked epoch. Short-term priming would manifest as a predisposition for the eyes to land on objects of the target color over the non-target color in the case of a repeat trial, and the opposite for a switch trial. Long-term priming would similarly manifest as a predisposition for the eyes to land on objects of the target color over the non-target color in the case of a bias-colored target trial, and the opposite for an other-colored target trial. As a dependent measure in either analysis, we computed this predisposition in the two conditions of interest (repeat and switch; bias and other), and these two measures were compared at each time point with *t* tests. The resulting time series of *t* values was used in a nonparametric permutation test (Maris and Oostenveld 2007) based on 1000 permutations, with threshold-free cluster enhancement (TFCE, Smith and Nichols, 2009) applied to every permutation. As a result, we identified the time points in the series at which either form of priming significantly biased gaze (p < 0.05).

## Results

### Behavioral results

#### Response time

**Fig. 2:**
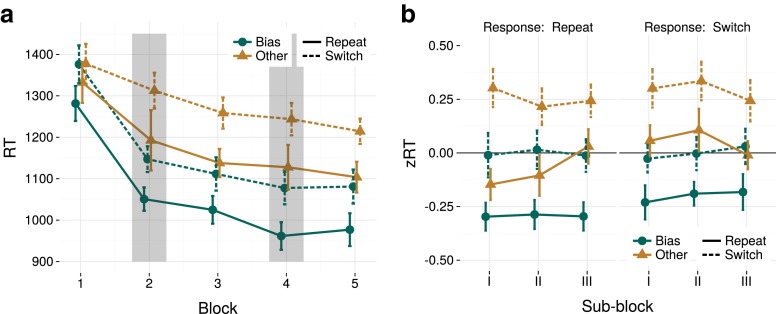
**a** Average response time (RT) as a function of experiment block, separately computed for target type (Bias or Other) and its relation with the previous trial (Repeat or Switch). In each block, shorter RTs are found for target repetitions than for switches (short-term priming). After the bias blocks, which are indicated by shaded *areas*, bias-color targets also yield faster RTs than other-colored targets (long-term priming). **b** z-scored RT of trials from Block 3 and 5, corrected for a priori color biases (see text for details), plotted across sub-blocks and separated for trials with a response repetition versus a response switch. This figure again shows the long- and short-term feature priming effect, and illustrates that the long-term priming effect does not attenuate across sub-blocks. In addition, it depicts a response priming effect: repeated responses yield shorter RTs than response switch trials. In these and all subsequent graphs, *error bars* (or shaded *error ribbons*) reflect Cousineau-Morey 95 % confidence intervals (Baguley 2011)

Figure 2a shows clear long- and short-term priming effects on RTs. Throughout the experiment, target color repetition resulted in faster RTs than switches. In the biased blocks (2 and 4), bias-color trials were processed faster than other-color trials. Bias-color trials remained faster than other-color trials in the neutral blocks 3 and 5, suggesting long-term priming of the bias color.

The statistical analyses (on zRT, as depicted in Fig. 2b) confirmed short- and long-term priming, and provided moderate evidence for an effect of response priming (*BF*_*S*_>1000,*BF*_*L*_>1000,*BF*_*R*_=6.12, respectively). Long-term priming did not attenuate over the course of a neutral block, which was supported by strong evidence against an interaction between long-term priming and sub-blocks (1/*BF*_*L*:*B*_=37.8 ). This is consistent with our earlier results, and suggests that the long-term priming effect cannot be explained as a cumulation of short-term priming effects. To further support this interpretation, we investigated higher-order priming effects, i.e., the amount of facilitation exerted by a repetition of trial *n*−2,*n*−3,.... As with earlier explorations (Maljkovic and Nakayama 1994; Maljkovic and Martini 2005), we found that short-term priming effects in this task dissipated within approximately five trials (depicted in Fig. S1). We did not find strong evidence to either confirm or reject a possible interaction between short-term priming and response repetition (1/*BF*_*S*:*R*_=1.37).

The posterior inferred from the full model revealed similar effect sizes for short- and long-term priming (*M* = 0.26,*CI* = [0.22,0.31];*M* = 0.25,*CI* = [0.21,0.30], respectively), and a smaller effect of response priming (*M* = 0.06,*CI* = [0.02,0.11]).

#### Subjective bias experience

Like in our original study, we rescaled subjective target bias estimates to a scale from -1 (only experienced other color trials) to 1 (only experienced bias color trials), and subjected these estimates to a one-sided Bayesian *t* test. This test provided moderate evidence that overall, participants correctly estimated that there had been a bias manipulation (*BF*_*δ*>0,*δ* = 0_=3.9). However, there was reasonable variability across participants (*M* = 0.13, *SD* = 0.28), who were almost evenly distributed between those who were aware of the bias (with estimates higher than 0, *N* = 13) and those who were not (*N* = 12). We therefore added an interaction term *A*:*L* to the full model that could account for a modulation of the long-term priming effect based on whether or not a participant was aware of the bias. The extended full model was not supported in favor of the model without this term, with 1/*BF*_*L*:*B*_=37.8. Moreover, even when accounting for any variation in long-term priming across people who were aware and people who were not, the extended model still showed overwhelming evidence for a main effect of long-term priming with *BF*_*L*_>1000. A graph depicting the long- and short-term priming effects for “Aware” compared to “Unaware” participants is given in Supplementary Fig. S2.

One could argue that only RTs from the last block should be considered in this analysis, as it is the block closest in time to the assessment of participants’ subjective experience. Repeating the analysis with this consideration yielded very similar outcomes (1/*BF*_*L*:*B*_=75.4; *BF*_*L*_>1000).

Despite the smaller power of these analyses, they show that the effect of the bias is not related to the awareness of it, suggesting that the long-term priming effect is not strategic in nature.

#### Accuracy

As was expected, no compelling evidence was found for any effects on accuracy. None of the terms in the top-down test were convincingly supported, each with 1/*BF*_*x*_>2.9. Of note, we confirmed from the posterior that the estimated mean effects of short- and long-term priming were both facilitatory (*M* = −0.005 and *M* = −0.001 respectively), and that these forms of priming thus did not reflect a speed-accuracy trade-off. In these analyses, accuracy was not corrected for a priori color biases because it is at the trial-level a discrete, factorial variable.

### Fixation results

#### Number of fixations per trial

**Fig. 3:**
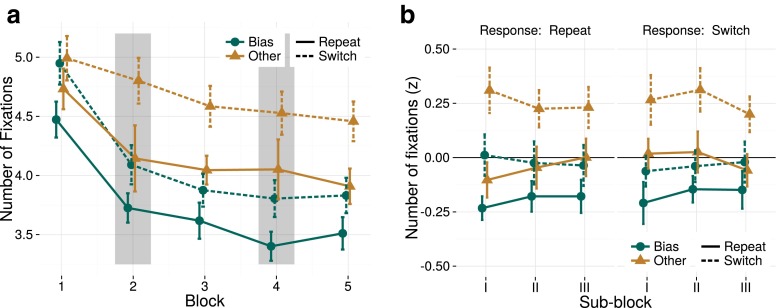
Conform Fig. 2, but using the number of fixations as a dependent measure. **a** Number of fixations recorded from the onset of the search display to the manual response (excluding the initial central fixation) as a function of target type (bias/other) and target repetition/switch relative to the previous trial. **b** Number of fixations across sub-blocks, z-scored and corrected for a priori color biases, plotted across sub-blocks and separated for response repetition- and switch-trials. Note the similarity between these data and the pattern of results for RT (Fig. 2): again, a difference was found between repetition and switch trials (short-term priming), and between bias color trials and other color trials (long-term priming). However, the repetition of responses does not seem to affect the number of fixations in a trial

Figure 3a depicts how the average number of fixations per trial changed over the course of the experiment, with respect to target repetition and target type. Overall, the number of fixations per trial decreased, indicating that participants became more efficient throughout the experiment. The pattern is qualitatively very similar to the evolution of RTs during the experiment. We investigated this similarity and found that a large amount of variance in RTs could be predicted by the number of fixations in a trial (mean *r*^2^=0.69,*SD* = 0.10), which suggests that participants heavily relied on eye movements to complete the task. Critically, less fixations per trial were needed when the target repeated. In Blocks 3 and 5, a clear difference has arisen between the bias- and other-color targets, suggesting that long-term priming affects the efficiency of the search process similarly to short-term priming.

The statistical analysis (on the z-scored number of fixations, Fig. 3b ) was in line with these observations, as again compelling evidence for short- and long-term priming effects was found, both with a *BF* > 1000. Additionally, we found strong evidence for an interaction between these two effects (*BF*_*S*:*L*_=16.8). No evidence was found for response repetition effects 1/*BF*_*R*_=9.7, or an interaction with short-term feature priming: 1/*BF*_*S*:*R*_=4.8. The main effect sizes were, again, very similar (*M* = 0.22,*CI* = [0.17,0.26] for short-term priming; *M* = 0.22,*CI* = [0.17,0.26] for long-term priming; their mean posterior difference differed less than 0.005). Their interaction was under-additive: long-term priming effects were larger on switch trials than on repetition trials (modulation of long-term priming effect on color repetition trials: *M* = 0.06,*CI* = [0.02,0.11]; the model interaction terms sum to zero, so the inverse held on switch trials). A practical explanation for this interaction, which was absent for all other dependent measures, could be a floor effect in the number of fixations: even on the fastest trials, there would usually be at least two fixations: a central one at trial onset, and one at or near the target.

#### Early color biases

**Fig. 4:**
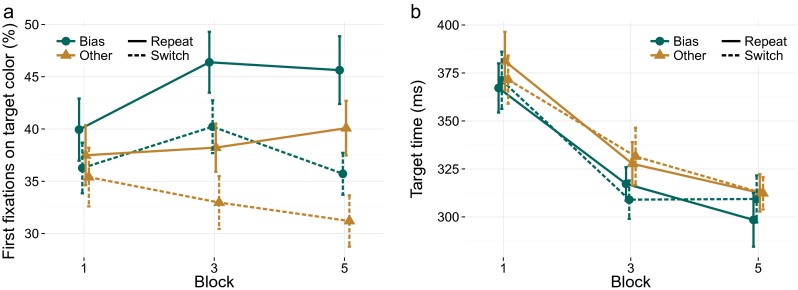
**a** The percentage of first fixations in each neutral block (1, 3, 5) that fall on the target color, as a function of whether the target color is the biased/other color, as well as a repetition/switch. Both short-term and long-term priming affect the first fixated color in a trial. **b** Average time between the onset of the final target fixation on a trial and the response, as a measure of the response phase. This phase is not affected by either short- or long-term feature priming effects (although this latency is affected by the response repetition, described in the text)

The analysis of the number of fixations strongly suggested that both long- and short-term priming modulated the search process. We investigated whether these priming effects would be reflected in what color the item had that was fixated first during search. To that end, we analyzed the proportion of trials where the first fixated color matched the target color. Like accuracy, this is at the trial level a factorial variable, and no a priori color correction was performed. A graph depicting this proportion over the course of the neutral blocks is provided in Fig. 4a.

The Bayesian ANOVA again confirmed effects of short- and long-term priming (both with a *BF* > 1000), and no other effects were found. In the statistical model, both terms again had strongly overlapping effect sizes (*M* = 0.07,*CI* = [0.05,0.09] for short-term priming; *M* = 0.06,*CI* = [0.04,0.08] for long-term priming). Effects of response repetition or an interaction between response and feature repetition were not supported (1/*BF*_*R*_=9.5;1/*BF*_*S*:*R*_=6.7 ).

#### Response phase effects

The analyses presented so far suggest that short- and long-term priming similarly affected visual search, by modulating the search process as early as on the first eye movement. Next, we analyzed whether either form of priming would affect the response phase of priming. To this end, we analyzed the time from the final target fixation onset to the response. This measure is comparable to target fixation duration as was, for example, used by Becker and Horstmann (2009), but it takes into account that the eyes might have shifted away from the target before a response key press was made. A graph for this measure is depicted in Fig. 4b.

The only term with positive evidence was response priming (*BF*_*R*_=9.5,*M* = 0.07,*CI* = [0.02,0.11]). No other terms were supported (all 1/*BF*_*x*_>3.5). Short- or long-term feature priming did not affect the response phase (1/*BF*_*S*_=9.6;1/*BF*_*L*_=7.9).

It can be argued that the total duration of target fixation constitutes a better measure of the ‘response phase’. This duration can be interpreted as indicative of the amount of time that evidence for either response could be accumulated. An analysis using this measure did not yield different outcomes. A clear response repetition effect was found, but other priming effects were not supported: (*BF*_*R*_>1000, 1/*BF*_*L*_=3.5. 1/*BF*_*S*_=5.5).

### Gaze results

The results from the analyses of fixation events revealed no differences between short- and long-term priming. Both seem to affect the search process early on, and have little to no effect on the response phase. To scrutinize these effects even further, we investigated how they developed over time, relative to the display onset, and relative to the response.

**Fig. 5:**
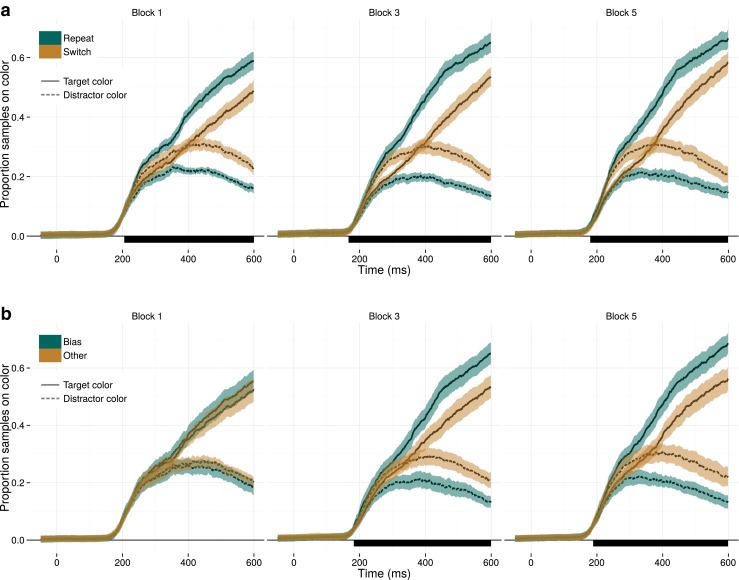
Long- and short-term priming throughout the search, within an epoch of 600 ms following display onset. **a** Throughout the experiment, the eyes fixate items sharing their color with the previous target color more than items with the other potential target color (here: distractor color). This difference arises at the first eye movement and persists at least throughout the entire epoch. **b** Long-term priming similarly biases participants’ gaze to items with the bias-target color from the first eye movement onwards. *Black bars under the graphs* mark timepoints with significant differences between the two colors, determined by the permutation test with TFCE

The time series with respect to the display onset are depicted in Fig. 5a comparing target repetition- and switch trials, and Fig. 5b comparing bias-colored targets to other-colored targets. From these graphs, it seems that participants made rather fast eye movements, as the proportion of fixations on either red and green stimuli began rising steeply at approximately 190 ms after display onset. Participants’ gaze is then subsequently drawn towards the target, illustrated by the steadily increasing proportion of fixations on the target color, and the gradual decrease of fixations on the distractor color. Critically, this is modulated by priming: Fig. 5a shows that in all blocks, participants’ gaze was biased towards the color that had been the target on the previous trial; Fig. 5b illustrates a similar search bias through long-term priming after the first block with a color bias.

These observations were corroborated by the statistical assessment of this time series. The one-sided permutation *t* test showed that short-term priming caused a significant (*p*<0.05) bias for the repeated target color as early as 185 ms (average onset of significance in all three blocks). For long-term priming, a similar difference onset was found, after 187 ms (average of blocks 3 and 5). Note that for both forms of priming this difference persisted throughout the entire epoch investigated here, up to 600 ms after display onset. A coarse estimate based on the time course of the first saccades suggests that for at least three gaze shifts priming affected the search process. Fig. 3 suggests that this is the majority of the entire search process.

**Fig. 6:**
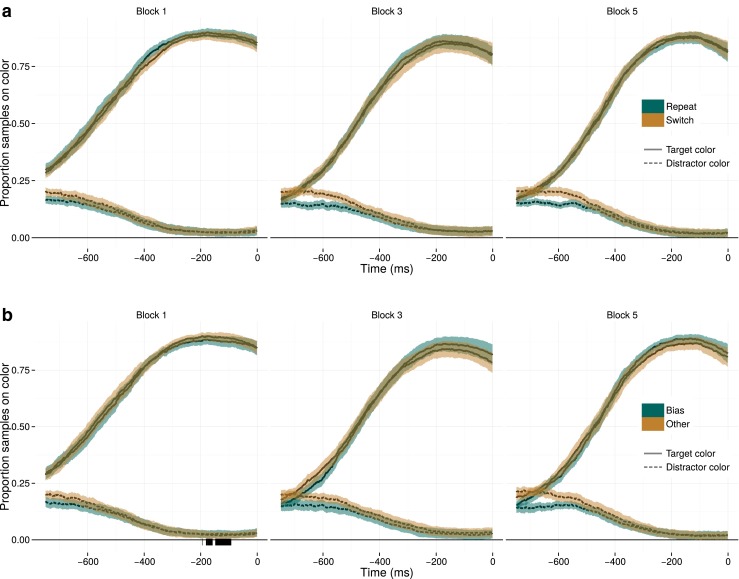
Data as in Fig. 5, but during a 750-ms epoch locked to the response. Long-and short-term priming do not seem to affect the response phase of a trial. If either form of priming were to affect the response phase, this would have been reflected by the ‘Switch’ curve (in **a**) and the ‘Other’ curve (in **b**) leading with respect to their counterparts. However, these curves follow identical time courses

The time-series analysis of the moments leading up to the response illustrates the lack of color priming effects on the response phase. This is depicted in Fig. 6. It is clear that in the time leading up to the response, the proportion of gaze samples that was on the target color increased. However, an effect on the response phase would imply that this curve would be leading for the unprimed color, as the time between landing on the target and formulating a response would be shorter for a primed color. Such an effect was not found by the statistical analysis. The one-sided *t* test did not reveal any differences for short-term priming, nor for long-term priming. Two very brief moments with a spurious significant difference between the biased and unbiased color were identified in block 1 (between -196 and -94 ms), but this cannot be interpreted as a form of color priming.

In the supplementary Fig. S4, the time-series analysis of the response epoch is depicted as a function of response repetition. That analysis again suggested that that unlike short- and long-term feature priming, response priming did affect the duration between fixating the target and producing a response, in line with the analyses presented above.

### Effects of position

Although not of immediate interest, we also explored whether target position priming affected search in this experiment. The analysis is discussed in more detail in the supplementary material, but we found strong evidence for positional priming, as shorter intertrial target distances led to shorter RTs. Like long- and short-term feature priming, positional priming effects appeared to manifest early on during the search, as we found strong evidence that the first eye movement tended to land close to the previous target location (Supplementary Fig. S5). Of note, we also found position biases of saccades that are unrelated to intertrial priming. Most participants showed a strong tendency to start searching each display at approximately the same location (Supplementary Fig. S6).

## Discussion

In this study, we have investigated what phases of visual search are affected by short- and long-term priming, and whether short- and long-term priming effects in visual search can be dissociated using eye movement recordings. We replicated the basic long-term priming results (Kruijne and Meeter 2015), finding that a bias block in which one target color is more frequent than another yielded strong, robust, and implicit facilitation of the search for the biased color. Here, we found that this effect was not dissociable from immediate intertrial repetition effects: both forms of priming modulate visual search through a bias of eye movements to the primed color. These modulations already affect the very first eye movements, from approximately 190 ms following stimulus onset onward. Conversely, short- and long-term priming were dissociated from response priming. Crucially, only response priming and response priming alone was found to affect the decision stage after the search was completed.

Research on priming in pop-out visual search has yielded strong, convergent evidence that short-term feature priming can affect the feedforward processing of a visual scene early on (Meeter and Van der Stigchel 2013; Bichot and Schall 2002; Maljkovic and Nakayama 1994; Tollner et al. 2008). Earlier research on priming in conjunction visual search (Becker and Horstmann 2009; Maljkovic and Nakayama 1996) has similarly suggested that intertrial priming changes the ‘attention-driving capacity’ of individual features, which also alludes to a modulation of the early feedforward signal. Here, we show that long-term feature priming exerts similar effects on overt attentional shifts: The earliest overt attentional shifts are biased towards features that have been presented as a target most often, similarly suggesting that long-term priming has enhanced the attention-driving capacity of stimuli with these features.

The hypothesis that long-term learning during a visual search task can affect the feedforward processing of stimuli with certain features is supported by various other studies. For example, participants can be trained to alter their *attentional set* by having them search for a target of one specific color among heterogeneously colored distractors. This will increase attentional capture by this particular color in a subsequent task that can be solved by simple singleton detection, rather than by attending specific features (Leber and Egeth, 2006; Leber et al., 2009; for a comparable observation, see Becker et al. (2014)). Neurophysiological support for long-lasting modulations of early visual processing was found in macaques by Bichot and Schall (1999b), who reported long-lasting enhanced responses in the Frontal Eye Fields for distractor stimuli that shared features with what had been the target of a conjunction search the previous day. Similar response enhancements (albeit only short-term) in Frontal Eye Fields were observed in a priming of (color) pop-out task, which was interpreted to reflect intertrial modulations of color signals in the ventral stream (Bichot and Schall 2002).

Given that both short-term priming of pop-out and long-term priming appear to affect visual processing in such a similar fashion, it may seem tempting to suggest that they are essentially equivalent. However, a clear empirical difference between the two speaks against such a generalization: long-term priming and similar effects are only found in conjunction- or feature search tasks. Although short-term priming is readily observed in singleton search tasks, such tasks do not yield long-term priming, even after prolonged ‘training’. That is: even when a singleton target remains constant or is heavily biased throughout an experimental block or even a session, little to no long-lasting effects are observed (Kruijne and Meeter 2015; Leber et al. 2009; Becker 2013; Bichot and Schall 1999b; Maljkovic and Martini 2005; Kruijne et al. 2015).

Previously, we proposed the following explanation for this dissociation between singleton search versus feature- and conjunction search (Kruijne and Meeter 2015). Since singleton search can be performed by simple bottom-up local comparisons, the absolute features may differ from trial to trial, but beyond the low-level comparison these features do not matter for the further processing of the search display. In conjunction- and feature search, on the other hand, local contrast is insufficient to complete the task. Instead, the absolute features of the target must be processed to determine whether a selected stimulus is a target. We proposed that only when task-relevant, these features are embedded in the memory traces that may later affect selection. This distinction relates to several theories that proposed that task-irrelevant features will not be encoded in memory and do not guide future attention (Turk-Browne et al. 2005; Hommel 2004; Thomson and Milliken 2013; Logan 2002).

A naive interpretation of guidance by memory traces would be that perception of the search display engenders retrieval of similar search trials. However, the apparent immediacy of the long-term priming effect found in this study suggests that this can not be the case. Retrieval takes time; for example, electrophysiological markers of memory retrieval are typically found only after at least 300 ms (Johnson Jr. et al. 1998) after onset of the memory probe (see Geyer et al., 2010, for a similar argument regarding contextual cueing). Note, however, that memory retrieval may already occur in preparation of the trial, affecting future visual processing before its onset. Recent studies have began to uncover how visual processing is modulated by memory retrieval, mediated by the hippocampus and the mediotemporal lobe (Hindy and Turk-Browne 2015; Turk-Browne et al. 2010), even when no visual information is present (Bosch et al. 2014). Recently, it was found that explicit mental imagery of search for a particular target can exert strong effects on subsequent visual search (Reinhart et al. 2015). It seems that implicit retrieval of past experience could bias attention in a similar fashion.

## Conclusions

The retrieval of past experience from long-term memory can help us guide our behavior when the future is uncertain. In the context of intertrial priming in visual search, it has been suggested that the effects of retrieval are limited to late, post-selectional processes (Huang et al. 2004; Lamy et al. 2010; Ásgeirsson and Kristjánsson 2011). Here, we found that long- and short-term priming have identical effects on visual search: they affect visual selection implicitly and immediately. We propose that retrieval of previous trials while anticipating the next one influences visual search. This provides a new perspective on how memory retrieval actually affects visual search.

## Electronic supplementary material

Below is the link to the electronic supplementary material.

(PDF 1.11 MB)

## Footnotes

1 This procedure is called ‘top-down’ because it begins with a high-level ‘full’ model after which each term is taken out one at a time. This is contrasted with a ‘bottom-up’ approach where one starts with a low-level ‘empty’ model that only includes the random effects term, where each term is added one at a time.
