# Supplementary material for: Implicit short- and long-term memory direct our gaze in visual search
Source: Atten Percept Psychophys. 2016 Jan 11;78:761–73. doi: 10.3758/s13414-015-1021-3 (PMC4819550; doi:10.3758/s13414-015-1021-3)
Supplement: Supplementary file 1 — (PDF 1.11 MB) [file 13414_2015_1021_MOESM1_ESM.pdf]

Graphs of additional measures

Figures S1 – S4 depict various figures of measures for which the corresponding statistical analyses are reported but the corresponding graphs are not included in the main text.

Positional Priming

We explored whether our task gave rise to intertrial position priming effects. This was investigated by exploring the effect of intertrial target distance and response times. A Bayesian regression analysis revealed strong evidence for such an effect ($BF_{\beta} > 1000$), which is also evident from Figure S5A.

Like long- and short-term feature priming, positional priming affects the first eye movement. We computed the distance from the first fixated location in a display to the target distance on the last trial. This was compared to the distance to the target ten trials ago, which was interpreted as a baseline with the assumption that positional priming effects should have long wained after ten trials (Martini, 2010). Indeed, a one-sided bayesian t-test indicated strong evidence that this distance was shorter than baseline ($BF_{\delta<0} > 1000$), as is also illustrated by the density plot of these distances in Figure S5B.

Idiosyncratic position biases

The results of this study show that the direction of the first saccade in a display is strongly affected by short- and long-term feature priming, as well as by positional priming. Of note, we additionally observed a systematic bias in our data for the location of the first saccade, which we illustrate for each participant in Figure S6. We present no statistical analyses of this phenomenon because it is not of immediate interest for priming. However, this graph clearly illustrates that most participants have a strong tendency to start their search of the display at the same location on most trials. Such an idiosyncratic location bias is apparent in most, but not all participants. Across individuals, this preferred appears to vary widely. It seems that such idiosyncratic biases would attenuate any effects on the direction of the first eye movement, and that they therefore form a factor to be considered in any study of visual search.

References

Martini, P. (2010). System identification in priming of pop-out. *Vision Research*, *50*(21), 2110-2115. doi: 10.1016/j.visres.2010.07.024

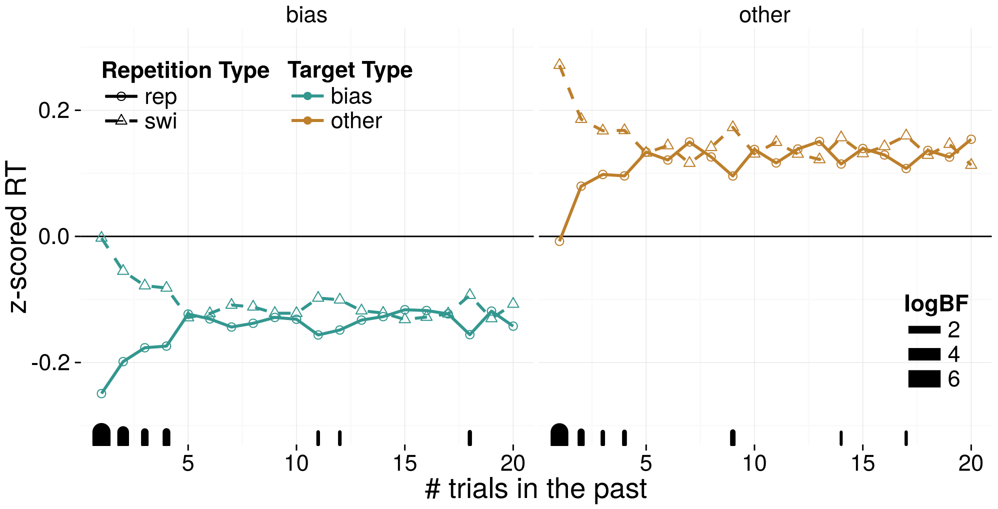

Figure S1. Higher order priming: the amount of facilitation exerted by by trial $n - k$ for $k \in [1 - 20]$, indicated by the difference in z-scored RT between repetition and switch trials. The marks at the bottom of the graph indicates the amount of evidence yielded by a one-sided Bayesian t-test for swi > rep. Marks are only plotted for ks with positive evidence, and the thickness of the tick-marks is indicative of the amount of evidence ($\log_{10}(\text{BF})$). The priming effect exerted by a single trial seems to dissipate within approximately five trials, as found in previous studies. After 5 trials, no more consistent evidence for priming effects is found.

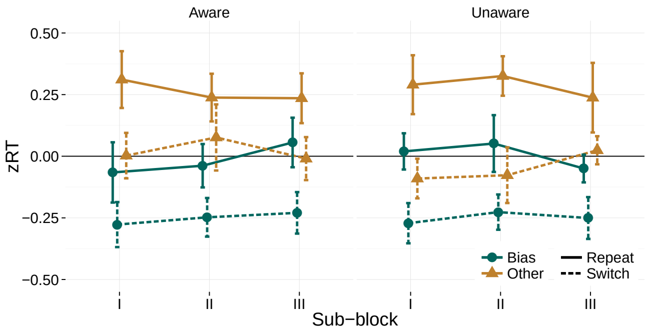

Figure S2. Long-term priming does not rely on whether participants had been aware of the bias. The effect is as prominent in participants who afterwards correctly estimated the direction of the bias ('aware') as in those that did not ('unaware')

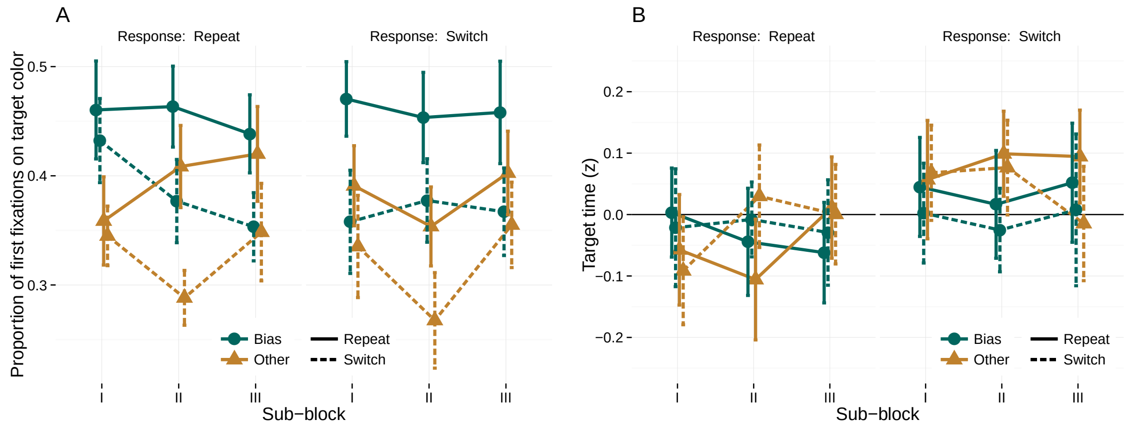

Figure S3. **A** Fixation biases induced by priming: the proportion of first fixations on an item with the target color, as modulated by short-, long- and response priming.
B Priming effects on target fixation duration: the effect of short-, long- and response priming on the z-scored, color-corrected time from fixating the target to making a response. (cf. Figure 4 in the main text).

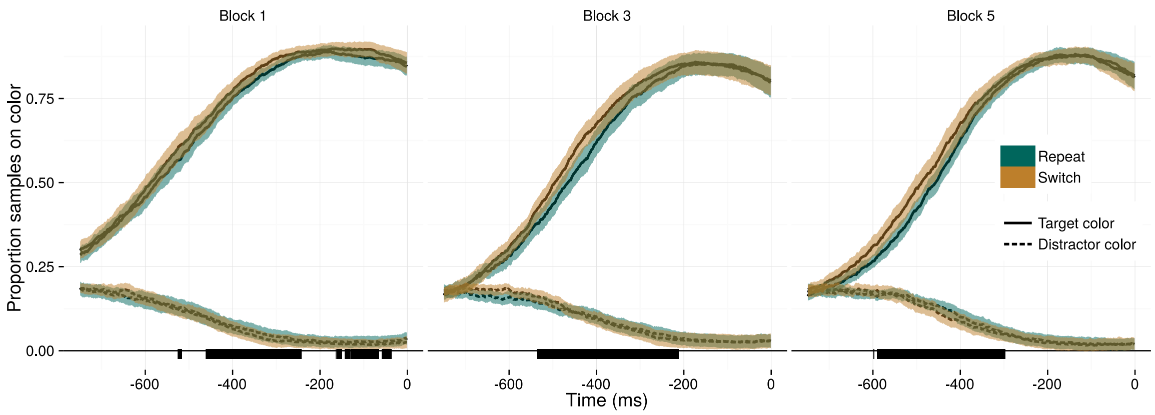

Figure S4. The proportion of samples on an item that has the target color or nontarget color, in the epoch 750 ms leading up to the response (cf. main text Figure 6), as a function of whether the response repeats or switches. Unlike long- and short- term feature priming, response priming affects the interval between fixating a target and the response. The curve for gaze samples on the target color during response-switch trials is leading with respect to the curve for response repetition trials. This indicates that on response switch trials, a longer time was necessary between fixating the target and producing a response. The black bars indicate a significant difference between the two types trials, based on a one-sided permutation test with TFCE (see main text for details)

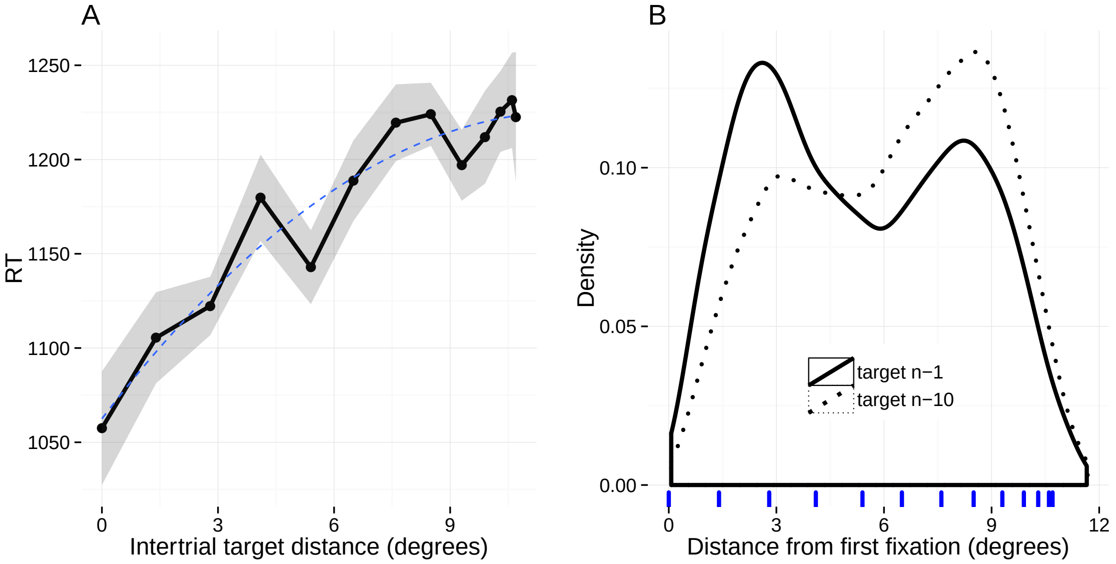

Figure S5. **A** Shorter intertrial target distances yield shorter RTs, signaling intertrial position priming. The blue dashed line is a quadratic fit to the data. **B** Density estimates of the distance from the first fixated location to the target on trial $n-1$ and to the target on trial $n-10$ (collapsed across all participants). The distribution is clearly skewed to lower distances for target $n-1$. The blue markers indicate the possible intertrial target distances, for reference.

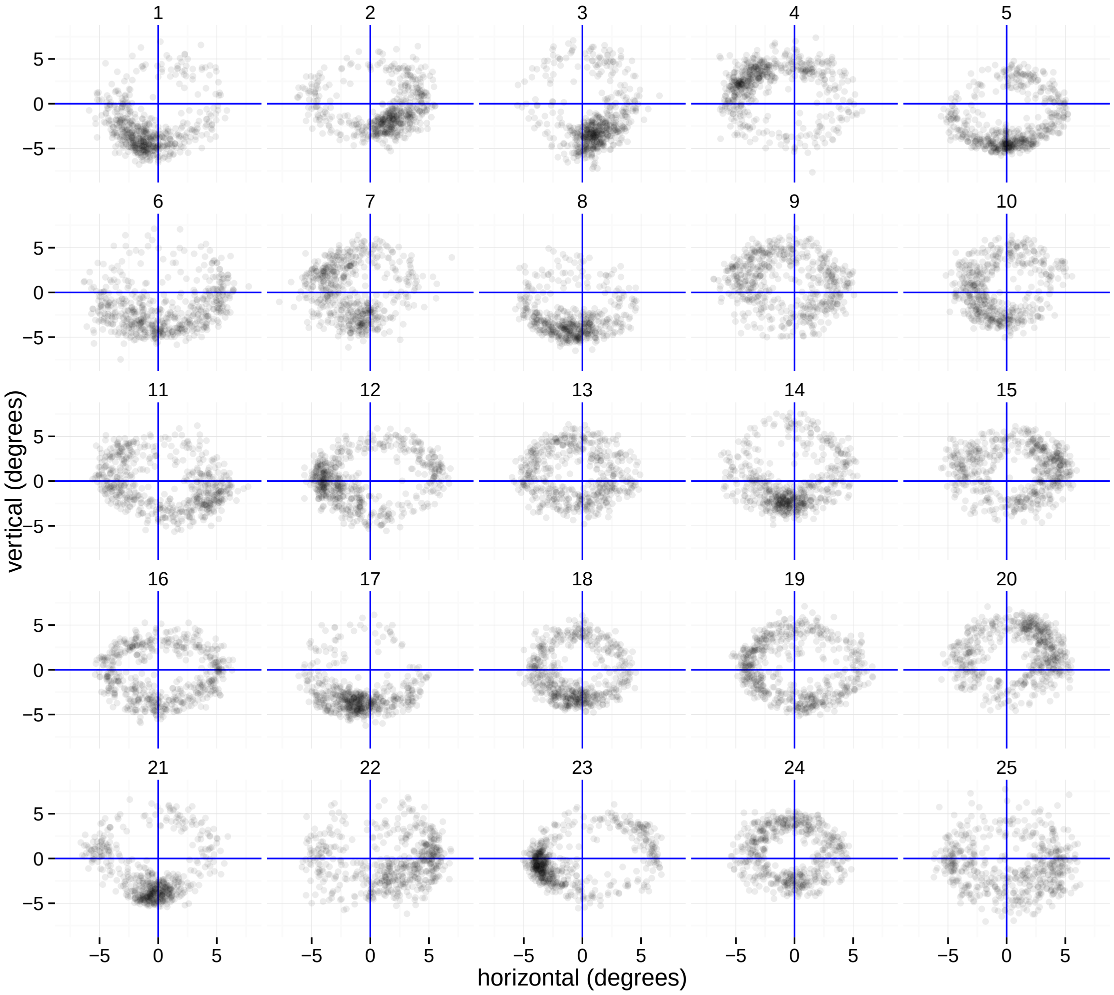

Figure S6. All landing positions of the first saccade made after display onset, separately plotted per participant
